# Extraction Optimization and Evaluation of the Antioxidant and α-Glucosidase Inhibitory Activity of Polysaccharides from *Chrysanthemum morifolium* cv. Hangju

**DOI:** 10.3390/antiox9010059

**Published:** 2020-01-09

**Authors:** Xiaoyan Hou, Xia Huang, Jianlong Li, Guangyang Jiang, Guanghui Shen, Shanshan Li, Qingying Luo, Hejun Wu, Meiliang Li, Xingyan Liu, Anjun Chen, Meng Ye, Zhiqing Zhang

**Affiliations:** 1 College of Food Science, Sichuan Agricultural University, Ya’an 625014, China; houxiaoyan106@outlook.com (X.H.);; 2 College of Biomass Science and Engineering, Sichuan University, Chengdu 610000, China; jlli999@foxmail.com; 3 College of Forest, Sichuan Agricultural University, Chengdu 610000, China

**Keywords:** *Chrysanthemum morifolium* cv. Hangju, polysaccharides, ultrasonic assisted extraction, antioxidant activity, α-glucosidase inhibitory activity

## Abstract

In order to evaluate the antioxidant and α-glucosidase activities of polysaccharides from *Chrysanthemum morifolium* cv. Hangju (CMPs), the response surface methodology was applied to optimize the parameters for extraction progress of CMPs by ultrasound, with heat reflex extraction (HRE) performed as the control. The difference in the physicochemical properties of polysaccharides obtained by the two methods were also investigated. The maximum yields (8.29 ± 0.18%) of polysaccharides extracted by ultrasonic assisted extraction (UAE) were obtained under the optimized conditions of ultrasonic power 501 W, extraction time 19 min, and ratio of liquid-to-raw material 41 mL/g. Polysaccharides extracted by UAE possessed lower protein contents (2.56%) and higher uronic acids contents (7.08%) and low molecular weight fractions than that by HRE. No significant differences were found in monosaccharide composition and Fourier transform infrared (FT-IR) spectra of polysaccharides extracted by UAE and HRE, while polysaccharides by UAE possessed stronger antioxidant and α-glucosidase inhibitory activities. Therefore, UAE was an efficient way to obtain CMPs.

## 1. Introduction

Oxidative stress, which can attack healthy cells and make their function and structure to be lost, is usually considered to be caused by reactive oxygen species (ROS) [1]. It is reported that more than 100 diseases such as Alzheimer’s disease, arteriosclerosis, and cancer are associated with oxidative stress [1,2]. Antioxidants are compounds or systems that can inhibit or delay the oxidation progress and play a significant role in antioxidant defense [3]. The functions of antioxidants such as lowering oxidative stress and reducing DNA and cell damage have been documented [1,2,3,4]. It has been stated that some exogenous antioxidants like vitamin C, vitamin E, and phenolics in plants can also perform the activity of endogenous antioxidative defense [5].

*Chrysanthemum morifolium* cv. Hangju is a plant belonging to the Compositae family [6]. It is a traditional Chinese herb, which is famous for its capacity to reinforce kidney, tonify spleen, and improve vision and has been used in folk medicine as a tea or drug for thousands of years [7,8]. Up to now, bioactive compounds such as lignans, phenolic glycosides, and flavonoids have been isolated from *C. morifolium* flowers [9,10]. Furthermore, these ingredients make the traditional herb exhibit numerous health benefits including antioxidant, anti-inflammatory, neuroprotective, and anti-HIV activity [10,11,12,13,14,15]. However, polysaccharides in *C. morifolium* flowers have rarely been studied. Only a few studies about *C. morifolium* polysaccharides (CMPs) have been reported [11,12,13,14]. Zheng et al. [13] studied a water soluble polysaccharide from *C. morifolium* and found that it showed excellent antioxidant activity. Moreover, a polysaccharide from the same material reported in another study exhibited anti-angiogenic activity. According to Tao et al. [11], polysaccharides from *C. morifolium* positively affected the short-chain fatty acids’ intestinal production and could prominently ameliorate colitis in rats.

Polysaccharides, presenting in almost all organisms, are important functional biological macromolecules because of their significant benefit to human health such as antioxidant, antidiabetic, immunopotentiation, antitumor, anti-inflammatory, and hypoglycemic activities [16,17,18]. More and more studies have shown evidence that polysaccharides have the capacity of scavenging free radicals and may be potential natural antioxidants. The biological activities of polysaccharides are mostly related to their physicochemical properties [19]. The conditions for extracting polysaccharides such as the extraction method, extraction time, or temperature and the ratio of solid-to-liquid are important due to their great impact on the structures and properties of polysaccharides. Heat reflux extraction (HRE) is the most common method for polysaccharides extraction. In general, HRE involves in a long extraction time and high temperature, causing the degradation of polysaccharides and decreasing their physiological activity [20]. Recently, ultrasonic assisted extraction (UAE) has become popular for its high efficiency, low consumption of energy, and high automation [20,21]. The cavitation effect of ultrasonic assisted extraction promotes the release of bioactive compounds from plant cells [22,23]. Moreover, a number of literature works suggested that polysaccharides extracted by UAE showed higher antioxidant activity than that of HRE [24,25,26]. Thus, UAE is a promising technique for polysaccharides’ extraction.

In the present work, the process of polysaccharides extraction by UAE was optimized using response surface methodology (RSM) with HRE as the control. Moreover, the physicochemical prosperities, antioxidant activity, and α-glucosidase inhibitory activity of polysaccharides obtained by the two methods were investigated and compared. The aim of the study is to reveal more information about the effect of extraction methods on the physicochemical properties and physiological activities of CMPs and provide a novel idea for full utilization of *C. morifolium* and improve its economic value.

## 2. Materials and Methods 

### 2.1. Plant Material and Chemicals

*C. morifolium* flowers were purchased from Tongxiang Shine Herb Health products Co., Ltd., (Jiaxing, China). The plant material was powdered and stored in a sealed bag until use. 1,1-Diphenyl-2-picrylhydrazyl radical (DPPH), trifluoroacetic acid (TFA), 1-phenyl-3-methyl-5-pyrazolone (PMP), trichloroacetic acid (TCA), butyl hydroxyanisole (BHA), 4-nitrophenyl-β-D-glucopyranoside (pNPG), and α-glucosidase (biological reagent, 50 U/mg) were purchased from Sigma-Aldrich (St. Louis, MO, USA). Acarbose, 1,10-phenanthroline, bovine serum albumin (BSA), and monosaccharide standards (glucose, fructose, rhamnose, galactose, xylose, arabinose) were purchased from J & K Scientific Co., Ltd. (Beijing, China). All other chemicals used were of analytical grade.

### 2.2. Extraction of Polysaccharides from C. morifolium

#### 2.2.1. Single Factor Analysis

The dried *C. morifolium* powder (5.0 g) was firstly refluxed with petroleum (50 mL) for 2 h and then 80% (*v*/*v*) ethanol (10 mL) for another 2 h to remove small molecular weight ingredients and lipids. The UAE was performed in an ultrasonic processor (JY99-IIDN, 1.8 kW, Xinzhi Biotechnology Co., Ltd., Ningbo, China). The influence of extraction time (5, 10, 15, 20, and 25 min), ultrasonic power (120, 240, 360, 480, and 600 W), and the ratio of liquid-to-solid (20, 30, 40, 50, and 60 mL/g) on the yield of CMPs was evaluated. The extracted polysaccharides (CMP-U i.e., *C. morifolium* polysaccharides obtained by UAE) were freeze dried and stored at −20 °C for further study.

#### 2.2.2. RSM Optimization

RSM was employed to obtain the optimum conditions based on the above work. A three level Box–Behnken design (BBD) with three variables (extraction time *X_1_*, ultrasonic power *X_2_*, and ratio of liquid-to-solid *X_3_*) were performed to optimize the extraction process. The complete design consisted of seventeen experimental points, and the variables and their levels with both coded and actual values are shown in Table 1. A quadratic polynomial model was fitted to correlate the response variable to the independent variables. The quadratic polynomial equation is generally as follows:

(1) $$Y\left(\%\right){=\alpha }_{0}\;+\;\overset{3}{\underset{i=1}{\sum }}\alpha_{i}X_{i}\;+\;\overset{3}{\underset{i=1}{\sum }}\alpha_{ij}X_{i}^{2}\;+\;\overset{2}{\underset{i=1}{\sum }}\overset{3}{\underset{j=i+1}{\sum }}\alpha_{ij}{\beta X}_{i}X_{j}$$

where *Y* (%) is the predicted response variable, *α_0_*, *α*_i_, *α_j_*, and *α_ij_* are the regression coefficients of the model, linearity, square, and interaction, respectively, and *X_i_* and *X_j_* are the independent variables. The data of this experiment were analyzed by multiple regression analysis through the least squares method. Analysis of variance (ANOVA) was applied to evaluate the observed data and the regression coefficients of linear, quadratic, and interaction terms, and the effects of the variables were generated and investigated.

The yield of polysaccharides (*Y*, %) is calculated as follows:

(2) $$Y(\%,w/w)=\frac{\mathrm{Weight}\;\mathrm{of}\;\mathrm{dried}\;\mathrm{crude}\;\mathrm{extraction}}{\mathrm{Weight}\;\mathrm{of}\;C.\;\mathrm{morifolium}\;\mathrm{powder}\;}\times 100$$

#### 2.2.3. Heat Reflux Extraction

HRE was carried out as the control to estimate the efficiency of UAE. The conditions for HRE involved an electric heater (SXKW, 500 mL, 0.3 kW, Beijing Ever Bright Medical Treatment Instrument Co., Ltd., Beijing, China) according to the optimized UAE. In brief, 5.0 g of pre-treated *C. morifolium* powder were mixed with 205 mL distilled water; the extraction temperature was set at 85 °C and the extraction time 2.5 h. The polysaccharides obtained by HRE (CMP-H) were freeze dried and stored at −20 °C for further use.

### 2.3. Determination of Chemical Composition and Molecular Weights

The contents of protein and uronic acids in CMPs were determined using Bradford’s method with BSA as a standard [27] and the carbazole-sulfuric acid method [28], respectively.

Molecular weight analysis of CMPs was performed using high performance gel permeation chromatography (HPGPC). In brief, the samples were first dissolved in deionized water. After passing through a 0.45 μm filter, the dissolved samples were applied to two Ultrahydrogel Linear columns (300 mm × 7.8 mm i.d.) in series; the columns were eluted with 0.1 M NaNO_3_. The flow rate was 0.9 mL/min, and the column temperature was 45 °C. The separation of CMPs was performed by a Waters 1525 high performance liquid chromatography (HPLC) system equipped with a Waters 2414 refractive index detector. A calibration curve was obtained using T-series dextran standards whose molecular weights ranged from 1 kDa to 2000 kDa.

### 2.4. Determination of Monosaccharide Composition

A method based on that of Wu et al. [29] was modified. The samples were first hydrolyzed with 4 M trifluoroacetic acid (100 °C, 6 h) to generate monosaccharides. Then, the solution was evaporated to dryness. The hydrolyzed polysaccharides (4 mg) were dissolved in 20 mL deionized water. Then, 400 μL of the solution were transferred to a centrifuge tube (2 mL) containing 200 μL of 0.3 M NaOH; 400 μL of 0.5 M methanol solution of 1-phenyl-3-methyl-5-pyrazolone (PMP) were added to the mixture and incubated at 70 °C for 0.5 h. After cooling to room temperature, 200 μL of 0.3 M HCl and 4 mL chloroform were sequentially added to the tube. The organic solvent layer (lower layer) was removed, and the procedure was repeated thrice. All the supernatants were collected, mixed, and filtered through a 0.22 μm membrane filter before HPLC analysis.

The monosaccharide compositions of CMPs were analyzed using an Agilent 1260 series HPLC system (Agilent, Palo Alto, CA, USA). A C_18_ column (4.6 mm × 50 mm, 1.8 μm; Zorbax Eclipse Plus, Agilent) was employed and eluted with a mixture of acetonitrile and phosphate buffer (20:80). The flow rate was 1 mL/min, and the UV detection was set at 250 nm at 30 °C. In addition, the injection volume was 10 μL, and the analysis time for each sample was 25 min. Six standard monosaccharides including galactose, arabinose, rhamnose, glucose, and fructose were used as the references after being derivatized by PMP.

### 2.5. Fourier Transform Infrared Spectrum Analysis

FT-IR analysis was employed to identify the organic functional groups of the CMPs by a spectrophotometer (Nicolet iS5, Thermo Fisher Scientific, Waltham, MA, USA). One milligram of CMPs extracted by different methods was ground with 100 mg dried KBr power, and the spectra were recorded at 4000 to 400 cm^−1^.

### 2.6. Antioxidant Activities of C. morifolium Polysaccharides

#### 2.6.1. DPPH Free Radical Scavenging Activity

The DPPH free radical scavenging activity was performed according to a previous method with a slight modification [13]. CMPs solutions were prepared in deionized water to a final concentration of 1.0, 2.0, 3.0, 4.0, and 5.0 mg/mL. One milliliter of the CMPs solutions was mixed with 3 mL DPPH solutions (0.1 mM in ethanol). After incubation for 30 min at 30 °C, the absorbance was recorded at 517 nm. Butyl hydroxyanisole (BHA) was used as the positive control. The DPPH scavenging activity was calculated as follows:

(3) $$\mathrm{DPPH}\;\mathrm{scavenging}\;\mathrm{activity}\;\left(\%\right)\;=\;\left(1-\frac{A_{s}}{A_{0}}\right)\;\times \;100$$

where *A_s_* is the absorbance of a mixture of DPPH and the CMP solution; *A*_0_ is the absorbance of the DPPH solution mixed with absolute ethanol. The results are also presented as an IC_50_ factor that represents the concentration of the sample that inhibits 50% of DPPH radicals.

#### 2.6.2. Hydroxyl Radical Scavenging Activity

The hydroxyl radicals scavenging assay was performed using a method described by Chen et al. [19] with a slight modification. CMPs solutions were prepared in deionized water to various concentrations (1.0, 2.0, 3.0, 4.0, and 5.0 mg/mL). Fifty microliters of CMPs solutions were firstly mixed with 50 μL of 3 mM 1,10-phenanthroline in a 96 well microtiter plate. Then, 50 μL of 3 mM FeSO_4_ and 50 μL of 0.01% aqueous H_2_O_2_ were added to each well. The reaction system was covered with aluminum foil and incubated at 37 °C for 1 h with shaking. The absorbance value was recorded at 536 nm. BHA was used as the positive control. The hydroxyl radical scavenging activity was estimated by the following equation:

(4) $$\mathrm{Hydroxyl}\;\mathrm{radicals}\;\mathrm{scavenging}\;\mathrm{activity}\;(\%)=\;[(A_{\mathrm{sample}}\;-\;A_{\mathrm{control}})/(A_{\mathrm{blank}}\;-\;A_{\mathrm{control}})]\;\times \;100$$

where *A_sample_* is the absorbance of the sample at 536 nm; *A_control_* is the absorbance of the control that contained a mixture of 1,10-phenanthroline, FeSO_4_, and H_2_O_2_; *A_blank_* is the absorbance of the blank solution in the absence of H_2_O_2_.

#### 2.6.3. Ferrous Chelating Activity

Ferrous chelating activity was measured using a method described before [18]. CMPs solutions were prepared in deionized water to different concentrations (1.0, 2.0, 3.0, 4.0, and 5.0 mg/mL). Then, 0.1 mL FeCl_2_ (2 mM), 0.15 mL ferrozine (5 mM), and 0.55 mL methanol were mixed with the samples (1.0 mL). The mixture was vortexed and incubated at room temperature for 10 min. The absorbance was recorded at 562 nm. EDTA was used as the positive control. The ferrous chelating activity of CMPs was calculated as follows:

(5) $$\mathrm{Ferrous}\;\mathrm{chelating}\;\mathrm{activity}\;\left(\%\right)\;=\;\left(1\;-\;\frac{A_{s}}{A_{0}}\right)\;\times \;100$$

where *A_s_* is the absorbance of the CMPs; *A*_0_ is the absorbance of the positive control.

#### 2.6.4. Reducing Power

A method based on that of Jing et al. [30] was modified to evaluate the reducing power of CMPs. CMPs solutions were prepared in deionized water to different concentrations (1.0, 2.0, 3.0, 4.0, and 5.0 mg/mL). Zero-point-one milliliters of K_3_ [Fe(CN)_6_] (1%, *w*/*v*, prepared in 20 mM PBS (pH 6.8)) were added in 0.1 mL of sample solutions, followed by incubating at 50 °C for 20 min. Then, 0.1 mL trichloroacetic acid (TCA, 10%, *w*/*v*) were added and vortexed. After centrifugation at 3000× *g* for 10 min, the supernatant (0.1 mL) was mixed with 0.1 mL of distilled water and 20 μL of FeCl_3_ (0.1%, *w*/*v*). After incubation at the temperature for 30 min, the absorbance was recorded at 700 nm. BHA was used as the positive control.

### 2.7. Alpha-Glucosidase Inhibition Assay

A method based on that of Wang et al. [31] was modified to evaluate the α-glucosidase inhibitory ability of CMPs. Alpha-glucosidase was prepared in PBS (0.1 M, pH 6.9) to a final concentration of 1 U/mL, and CMPs were prepared in deionized water to final concentrations of 1.0, 2.0, 3.0, 4.0, and 5.0 mg/mL. One hundred microliters of the Alpha-glucosidase solution were mixed with 50 μL of the sample, and the mixture was then incubated at 25 °C for 10 min in a 96 well plate. After that, 50 μL of 4-nitrophenyl-β-D-glucopyranoside (*p*NPG, 5 mM, prepared in 0.1 M PBS) were added, and the mixture was incubated again at 25 °C for 5 min. The absorbance was determined at 405 nm before and after the last incubation. Acarbose was used as the positive control. The α-glucosidase inhibitory activity can be expressed as follows:

(6) $$\mathrm{Inhibition}\;(\%)\;=\;(1\;-\;\Delta A_{\mathrm{sample}}/\Delta A_{\mathrm{control}})\;\times \;100$$

where *A_sample_* is the absorbance of the sample and *A_control_* is that of the control.

### 2.8. Statistical Analysis

Statistical analysis was performed using SPSS software (Version 20.0, IBM SPSS, Armonk, NY, USA). All the experiments were carried out three times, and the data are expressed as the means ± the standard deviation. Their statistical significance of difference was determined with Tukey’s multiple comparison test. A value of *p* < 0.05 was considered statistically significant.

## 3. Results and Discussion

### 3.1. Single Factor Assessment

#### 3.1.1. Effect of the Ratio of Liquid-to-Solid on the Yield of CMP

**Figure 1:**
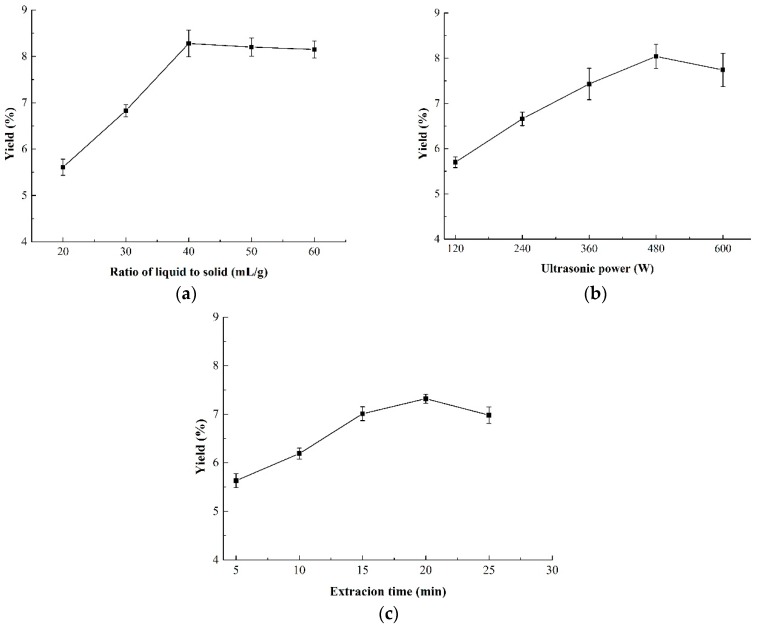
Effect of three independent variables on the yield of CMP-U. (**a**) Ratio of liquid-to-solid; (**b**) ultrasonic power; and (**c**) extraction time. CMP-U: C. morifolium polysaccharides obtained by ultrasonic assisted extraction.

The effect of the ratio of liquid-to-solid on the yield of CMPs is shown in Figure 1a. It was suggested that the yield of CMP increased significantly in the range of 20–40 mL/g and reached a peak of 8.28% at 40 mL/g. However, there was no obvious increase when the ratio of liquid-to-solid continued to rise above 40 mL/g. This meant that a higher ratio of raw material (>40 mL/g) resulted in no higher yield, indicating that the ratio of liquid-to-solid higher than 40 mL/g was not necessary.

#### 3.1.2. Effect of Ultrasonic Power on the Yield of CMP

The effect of ultrasonic power on the yield of CMP is shown in Figure 1b. It was obvious that the yield increased with the increasing ultrasonic power from 120 W to 480 W and reached a maximum of 8.04% at 480 W. Ultrasonic power higher than 480 W caused the decrease of CMP yield which, may be due to the reason that higher ultrasonic power would result in degradation of polysaccharides [32]. Thus, the suitable ultrasonic power for the BBD design was from 360 to 600 W.

#### 3.1.3. Effect of Extraction Time on the Yield of CMP

The yield of CMP under different extraction time is shown in Figure 1c. An obvious increase of CMP yield was observed from 5 to 20 min. However, the yield of CMP exhibited a decreasing trend when the extraction time further increased. It was reported that longer extraction time in the ultrasonic extraction might induce the degradation of polysaccharides and decrease the yield. The results were in correspondence with those of Maran et al. [33] and Guo et al. [24], both of whom declared that 20 min of ultrasonic treatment was sufficient enough for polysaccharides extraction. Therefore, 20 min of extraction time was chosen as the central point of the BBD design.

### 3.2. Extraction and Optimization of CMP by RSM

#### 3.2.1. Model Fitting

According to the single factor experiments, a total of seventeen runs of the BBD experiment was performed to optimize the UAE. Three independent variables including extraction time *X_1_*, ultrasonic power *X_2_*, and the ratio of liquid-to-solid *X_3_* were optimized, and the corresponding results are shown in Table 1. The final equation obtained in terms of coded factors is described as below:

(7) $$Y_{2}\;=\;8.25\;-\;0.21X_{1}\;+\;0.17X_{2}\;+\;0.31X_{3}-\;0.22X_{1}X_{2}\;+\;0.66X_{1}X_{3}\;+\;0.72X_{2}X_{3}\;-\;0.39X_{1}^{2}\;-\;0.83X_{2}^{2}\;-\;1.31X_{3}^{2}$$

ANOVA is shown in Table 2. The model F-value (133.87) and *p*-value (<0.0001) indicated that the model was significant. Furthermore, the adjusted determination coefficient value (*R_adj_^2^* = 0.9868) also confirmed the high significance of the model. The determination coefficient (*R^2^*) was 0.9942, indicating that only 0.58% of the total variance was not explained by the model. In addition, the linear coefficients (*X_1_*, *X_2_*, and *X_3_*), interaction terms (*X_1_X_2_*, *X_1_X_3_*, and *X_2_X_3_*) and quadratic terms (*X_1_^2^*, *X_2_^2^*, and *X_3_^2^*) were significant (*p* < 0.05), implying that these items could significantly affect the yield of CMP. The *p*-value (0.2312) and F-value (2.20) of the lack of fit for the model demonstrated that it was not significant relative to the pure error, which indicated that the model was credible. The low value of the coefficient of variation (CV, 1.68%) indicated the similarity between predicted and experimental values, suggesting that the model had a high degree of reliability.

#### 3.2.2. Response Surface Analysis and Verification of the Model

**Figure 2:**
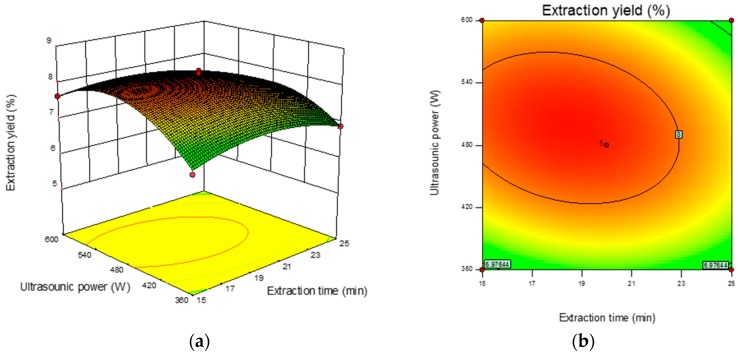

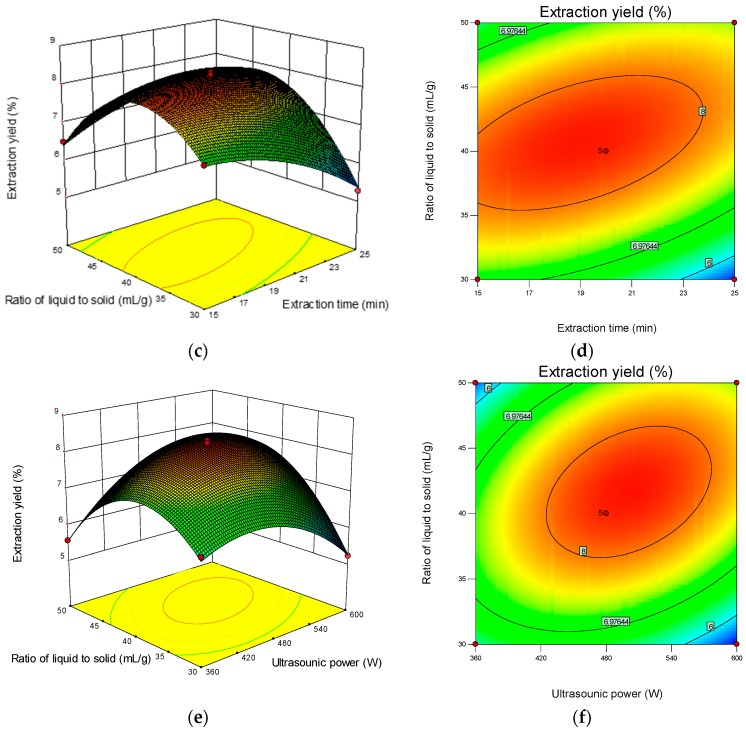
Response surface plots (left) and contour plots (right) showing the interactive effects of different variables on the yield of CMP-U. (**a**,**b**) Ultrasonic power and extraction time; (**c**,**d**) ratio of liquid-to-solid and extraction time; (**e**,**f**) ratio of liquid-to-solid and ultrasonic power.

The 3D response surface and 2D contour plots revealed the interaction among the variables and the response. As shown in Figure 2b, the contour plot was elliptical, suggesting that the mutual interactions between extraction time and ultrasonic power were significant. A similar trend was found for extraction time and the ratio of liquid-to-solid (Figure 2d) and ultrasonic power and the ratio of liquid-to-solid (Figure 2f). The optimum parameters obtained from the above experiment were as follows: extraction time 18.90 min, ultrasonic power 501.36 W, and ratio of liquid-to-solid 41.13 mL/g. Under the optimized condition, the maximum predicted yield of CMP was 8.31%. The verification assays were conducted under the optimized conditions, and the actual extraction yield was 8.29 ± 0.18%, which was in correspondence with the predicted value.

In the present work, HRE was conducted to evaluate the superiority of UAE in extracting polysaccharide from *C. morifolium*. CMP obtained by HRE (7.25 ± 0.10%, data not shown) at the optimized conditions was lower than that of UAE. Moreover, the UAE procedure took less time (19 min) compared with HRE (2.2 h). Power consumption of the two methods was 0.57 kWh for UAE and 0.66 kWh for HRE. Therefore, UAE was a less time consuming and more efficient way to extraction CMPs.

### 3.3. Physicochemical Properties of CMPs

As shown in Table 3, the extraction yields of CMP-U (8.29 ± 0.18%) were higher than those of CMP-H (7.25 ± 0.10%) due to mechanical fluctuation and ultrasonic cavitation effect [34]. Besides, CMP-U had a higher content of uronic acid than CMP-H, which was determined to be 7.08 ± 0.25% and 1.61 ± 0.10%, respectively. It was reported that uronic acid is of great importance to the biological activities of polysaccharides [35]. The protein content in CMP-U and CMP-H was 2.56 ± 0.08% and 3.36 ± 0.09% according to the Bradford method. Therefore, in terms of extraction yield, UAE is a method that used less power and had shorter time and lower temperature. He et al. [36] found that *Polyporus umbellatus* polysaccharides with higher uronic acids content exhibited higher antioxidant activity. In another work, it was reported that the amount of uronic acids could influence the antioxidant capacity and free radicals scavenging activity of Astragalus membranaceus polysaccharides [37].

**Figure 3:**
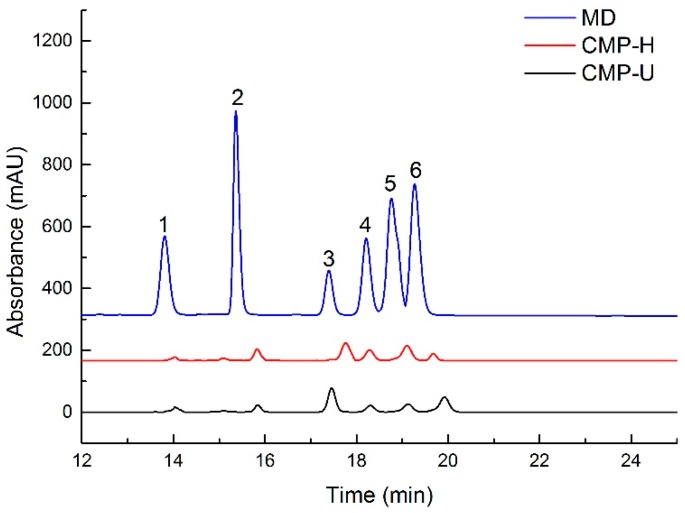
HPLC chromatogram of monosaccharides in CMPs. MD: mixed standard of monosaccharides; 1: fructose; 2: rhamnose; 3: glucose; 4: galactose; 5: xylose; 6: arabinose; CMP-H: *C. morifolium* polysaccharides obtained by hot reflux extraction; CMP-U: *C. morifolium* polysaccharides obtained by ultrasonic assisted extraction.

HPLC results indicated that both CMP-U and CMP-H contained galactose, rhamnose, glucose, and fructose, but in different proportions (shown in Table 3 and Figure 3). The monosaccharide composition showed that glucose (76.40% and 69.08, respectively) was the dominant sugar for CMP-U and CMP-H. Moreover, the molar ratios of glucose, fructose, rhamnose, galactose, xylose, and arabinose in CMP-U were 1:0.011:0.04:0.065:0.113:0.184, and those in CMP-H were 1:0.007:0.052:0.095:0.214:0.045.

### 3.4. Molecular Weight Distribution of CMPs

**Figure 4:**
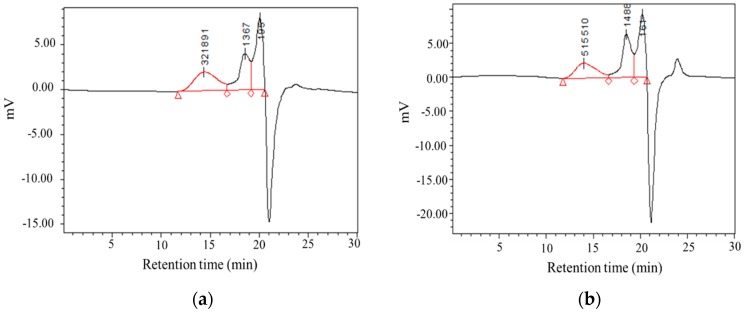
Gel permeation chromatography (GPC) spectrums of *C. morifolium* polysaccharides. (**a**) CMP-H; (**b**) CMP-U. CMP-H: *C. morifolium* polysaccharides obtained by hot reflux extraction; CMP-U: *C. morifolium* polysaccharides obtained by ultrasonic assisted extraction.

The molecular weights and monosaccharide composition of CMP samples were determined by HPGPC and HPLC. As is shown in Table 4 and Figure 4, both CMP-H and CMP-U showed three peaks in size exclusion chromatography. Moreover, the low molecular weight fractions in CMP-U (73.72%) were higher than those of CMP-H (62.56%). This could be attributed to the degradation of CMPs caused by ultrasonic power, which was correspondent with previous literature [24,38].

### 3.5. FT-IR Analysis

**Figure 5:**
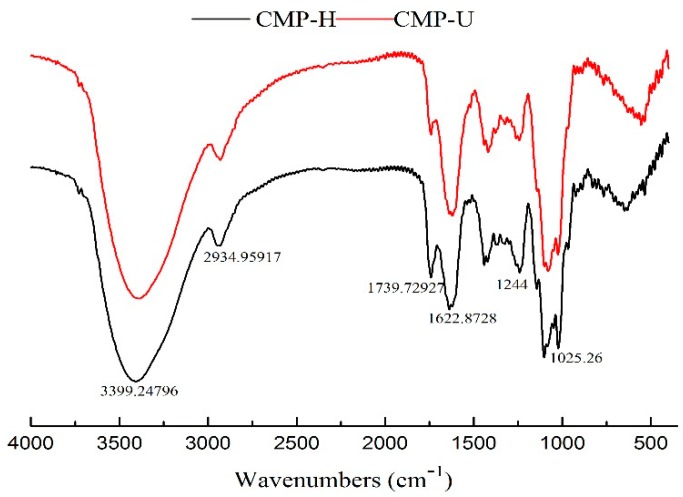
Fourier transform infrared (FT-IR) spectra of CMP-H and CMP-U. CMP-H: *C. morifolium* polysaccharides obtained by hot reflux extraction; CMP-U: *C. morifolium* polysaccharides obtained by ultrasonic assisted extraction.

The FT-IR spectra of CMP-U and CMP-H are shown in Figure 5. No significant difference was observed between the spectra of the two CMPs. The peaks at ~3400 cm^−1^ and 2930 cm^−1^ were the stretching vibration of the hydroxyl group and C–H bond, respectively [39]. The absorption band near 1740 cm^−1^ was the stretching vibration of C=O [40]. The absorption peaks near 1600–1622 cm^−1^ was the stretching vibration of the carboxylate anion, indicating the presence of uronic acids [24]. The absorption in the range 1244–1000 cm^−1^ could be attributed to the stretching vibration of C–O–C or C–OH bonds of a pyranose ring, which comprises the characteristic absorbance of polysaccharides [31].

### 3.6. Antioxidant Activity Assay of CMPs by Different Extraction Methods

#### 3.6.1. Scavenging Activity of DPPH Radicals

**Figure 6:**
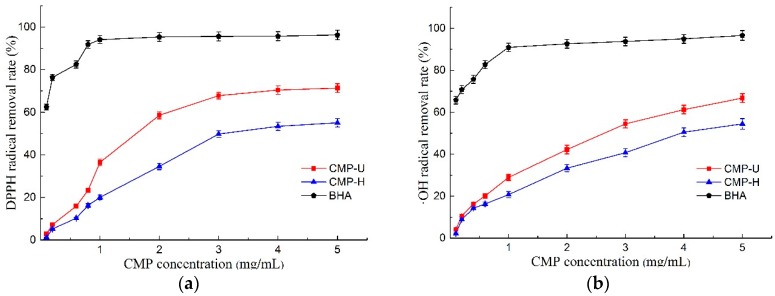

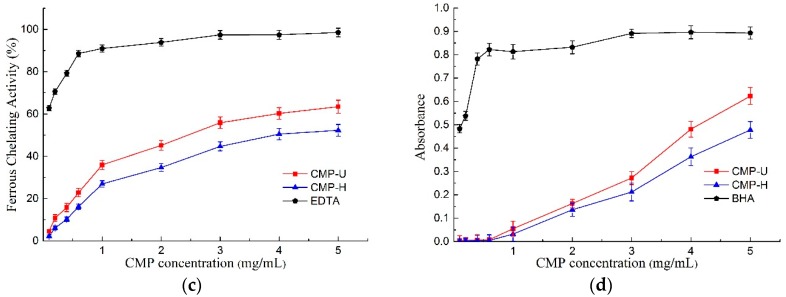
Antioxidant activities of CMP-H and CMP-U. (**a**) DPPH free radical scavenging activity; (**b**) hydroxyl radical scavenging activity; (**c**) ferrous chelating activity, and (**d**) total reducing power. CMP-H: *C. morifolium* polysaccharides obtained by hot reflux extraction; CMP-U: *C. morifolium* polysaccharides obtained by ultrasonic assisted extraction.

DPPH free radical scavenging activity has been widely used as an important index to evaluate the antioxidant activity of natural products [41]. As shown in Figure 6a, both CMP-U and CMP-H exhibited scavenging activity of the DPPH free radical in a dose-dependent manner. Moreover, the DPPH scavenging ability of CMP-U was higher than that of CMP-H at different concentrations. It was suggested that UAE may be an effective method to obtain CMPs with excellent antioxidant activity. Furthermore, the IC_50_ values of DPPH free radical scavenging activity for CMP-U and CMP-H were 1.850 mg/mL and 3.587 mg/mL, which were higher and hence worse than that of BHA (0.047 mg/mL). 

#### 3.6.2. Scavenging of Hydroxyl Radicals

The scavenging activities of hydroxyl radical play a significant role in protecting living cells due to the bad effects of the free radicals such as causing cancer, and damaging DNA or proteins [3]. The scavenging activities of CMP-U and CMP-H are shown in Figure 6b. Similar to DPPH scavenging activity, the hydroxyl radical scavenging ability of CMPs also exhibited a good concentration dependent manner. It was obvious that CMP-U possessed higher hydroxyl radical scavenging activity than CMP-H. Moreover, the hydroxyl radical scavenging activity of CMP-U and CMP-H reached a peak value of 66.77% and 54.46% at 5.0 mg/mL. The IC_50_ values of hydroxyl radical scavenging activity for CMP-U and CMP-H were 2.612 mg/mL and 4.236 mg/mL, respectively. It was proposed that the hydrogen or electron abstraction mechanism might be attributed to the hydroxyl radical inhibition of polysaccharides [42]. According to Fan et al. [43], stronger hydroxyl radical inhibition activity might be related to higher contents of uronic acids. Based on this report, a higher content of uronic acids in CMP-U contributed to its higher hydroxyl radical scavenging activity. 

#### 3.6.3. Ferrous Chelating Activity

The ferrous chelating activity of CMPs is presented in Figure 6c. Both polysaccharides displayed ferrous chelating activity in a concentration dependent manner, and CMP-U showed greater ferrous chelating activity. The IC_50_ values of ferrous chelating activity for CMP-U and CMP-H were 2.523 mg/mL and 3.982 mg/mL. At the concentration of 5.0 mg/mL, the ferrous chelating activity of CMP-U and CMP-H reached the peak of 63.47% and 52.29%, respectively. It was reported that the observed chelating activity of polysaccharides was likely related to the content of galactose in the polysaccharide. It is interesting to note that CMP-U possessed higher galactose content than CMP-H, and this observation was consistent with previous reports [18,44].

#### 3.6.4. Total Reducing Power

The antioxidant can reduce the reactive groups to more stable species by donating electrons to them [3]. The total reducing power of an antioxidant compound essentially indicates whether it is a good electron donor and is related to its antioxidant activity [45]. In this study, greater ultraviolet absorption was indicative of better reducing power [46]. As can be seen from Figure 6d, the total reducing power increased in a dose dependent manner according to CMP concentration. Moreover, the reducing power of CMP-U was higher than that of CMP-H. Reducing power was reported to be associated with reductones, which were proposed to react with precursors of peroxide [47].

### 3.7. Alpha-Glucosidase Inhibitory Activities

**Figure 7:**
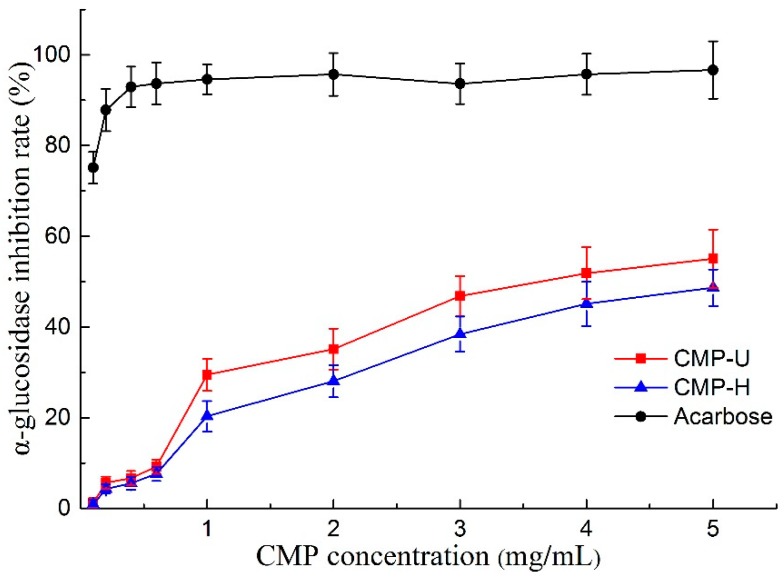
α-glucosidase inhibitory effect of CMP-U and CMP-H. CMP-H: *C. morifolium* polysaccharides obtained by hot reflux extraction; CMP-U: *C. morifolium* polysaccharides obtained by ultrasonic assisted extraction.

Alpha-glucosidase is a key enzyme associated with the digestion of carbohydrates in the small intestine, and the inhibition of α-glucosidase can delay the breakdown of starch, keeping the blood glucose at low levels [48]. Therefore, α-glucosidase inhibitors are key factors for the treatment of type II diabetes. As shown in Figure 7, both CMP-U and CMP-H exhibited α-glucosidase inhibitory activity in a concentration dependent manner. The IC_50_ values of CMP-U and CMP-H were 3.606 and 4.854 mg/mL, higher than that of acarbose (0.01 mg/mL). It was suggested that CMP-U had better α-glucosidase inhibition activity than CMP-H when the concentration was higher than 1.0 mg/mL (*p* < 0.05), showing a similar result to those from antioxidant assays. The observation corresponded well with previous studies in which positive correlations were found between antioxidant activities of polysaccharides from oolong tea and their α-glucosidase inhibition activities [31,49]. The discovery of natural anti-diabetic agents is becoming more and more popular due to the side effects of synthetic anti-diabetic drugs. Polysaccharides extracted from several plants have been reported to exhibit α-glucosidase or α-amylase inhibition activity [18,31,48]. However, there has been no report on α-glucosidase inhibition activity of *C. morifolium* polysaccharides to the best of our knowledge. Therefore, the findings in our study indicate that UAE is an efficient way to obtain CMPs exhibiting inhibitory potential against α-glucosidase. In addition, CMPs may be used as functional food additives that are beneficial for diabetic patients.

### 3.8. Relationship between Physicochemical Properties of CMPs and Their Antioxidant Activity or α-Glucosidase Inhibition Activities

According to previous literature, the antioxidant activity of polysaccharides was affected by a combination of many factors, including molecular weight, structure, monosaccharide composition, and conformation [50]. In our present work, it was suggested that CMP-U with lower protein content and higher uronic acid content exhibited stronger scavenging activity of DPPH radicals and hydroxyl radicals, ferrous chelating activity, reducing power, and α-glucosidase inhibitory activity. The result was consistent with previous reports. Uronic acids were considered to play a significant role in the antioxidant activity of polysaccharides; a higher content of uronic acids in polysaccharides tended to exhibit stronger antioxidant activity [36]. Moreover, the number of low molecular weight fractions of CMP-U was higher than that of CMP-H, contributing to the stronger antioxidant activity and α-glucosidase inhibitory activity of CMP-U. Dong et al. [51] reported that the higher the number of low molecular weight fractions, the higher the antioxidant activity of polysaccharides. However, the exact mechanism by which these physicochemical properties affect the antioxidant activity or α-glucosidase inhibition activity is unclear, and it will be deeply investigated in our further work.

## 4. Conclusions

In our present work, ultrasonic technology was used to extract polysaccharides from *C. morifolium*. HRE was employed as a control to evaluate the efficiency of UAE. The best conditions of UAE optimized by response surface methodology were ultrasonic power 501 W, extraction time 19 min, and ratio of liquid-to-raw material 41 mL/g. Under these conditions, the yield of CMP-U was 8.29 ± 0.18%. Compared to HRE, the extraction yield of UAE was increased, the extraction time was greatly shortened, and the power consumption was lower. Investigation of physicochemical properties indicated that polysaccharides extracted by UAE had lower content of protein and higher content of low molecular weight fractions and uronic acids. Meanwhile, CMPs by the two methods showed similar monosaccharide composition and Fourier transform infrared (FT-IR) spectra. Moreover, polysaccharides extracted by UAE exhibited higher antioxidant and α-glucosidase inhibition activity. All these results indicated that UAE was an efficient way to obtain *C. morifolium* polysaccharides with high antioxidant and α-glucosidase inhibitory activity. In addition, CMPs could be used as natural food additives with antioxidant and α-glucosidase inhibition activities in the food industry.

## Figures and Tables

**Table 1:** Box–Behnken design matrix and the response values for the yield of CMPs by UAE.

Run	Levels on Independent Factors			Extraction Yield (%)
	*X_1_* (min)	*X_2_* (W)	*X_3_* (mL/g)	
1	20 (0)	480 (0)	40 (0)	8.29
2	25 (1)	480 (0)	30 (−1)	5.28
3	20 (0)	480 (0)	40 (0)	8.36
4	25 (1)	360 (−1)	40 (0)	6.88
5	20 (0)	600 (1)	30 (−1)	5.23
6	20 (0)	360 (−1)	30 (−1)	6.46
7	20 (0)	480 (0)	40 (0)	8.20
8	15 (−1)	480 (0)	50 (1)	6.50
9	20 (0)	360 (−1)	50 (1)	5.56
10	20 (0)	600 (1)	50 (1)	7.22
11	25 (1)	480 (0)	50 (1)	7.31
12	15 (−1)	600 (1)	40 (0)	7.63
13	15 (−1)	360 (−1)	40 (0)	6.75
14	20 (0)	480 (0)	40 (0)	8.29
15	25 (1)	600 (1)	40 (0)	6.89
16	20 (0)	480 (0)	40 (0)	8.11
17	15 (−1)	480 (0)	30 (−1)	7.13

***X_1_***: extraction time; ***X_2_***: ultrasonic power; ***X_3_***: ratio of liquid-to-solid.

**Table 2:** Analysis of variance (ANOVA) testing the fitness of the regression equation.

Source	Sum of Squares	Df	Mean Square	F-Value	*p*-Value
Model	17.03	9	1.89	133.87	<0.0001
X_1_	0.34	1	0.34	24.07	0.0017
X_2_	0.22	1	0.22	15.40	0.0057
X_3_	0.78	1	0.78	54.81	0.0001
X_1_X_2_	0.19	1	0.19	13.38	0.0081
X_1_X_3_	1.77	1	1.77	125.11	<0.0001
X_2_X_3_	2.09	1	2.09	147.68	<0.0001
X_1_^2^	0.63	1	0.63	44.71	0.0003
X_2_^2^	2.87	1	2.87	202.68	<0.0001
X_3_^2^	7.20	1	7.20	509.09	<0.0001
Residual	0.099	7	0.014		
Lack of fit	0.062	3	0.021	2.20	0.2312
Pure error	0.037	4	9.350 × 10^−3^		
Cor total	17.31	16			
R^2^	0.9942				
Adjusted R^2^	0.9868				
CV %	1.68				

**Table 3:** Physicochemical properties of *C. morifolium* polysaccharides obtained by UAE and HRE.

Items	CMP-U	CMP-H
Yield (%)	8.29 ± 0.18 ^a^	7.25 ± 0.10 ^b^
Protein content (%)	2.56 ± 0.08 ^b^	3.36 ± 0.09 ^a^
Uronic acid (%)	7.08 ± 0.25 ^a^	1.61 ± 0.10 ^b^
Constituent monosaccharides and molar ratios		
Glucose	1	1
Fructose	0.011	0.007
Rhamnose	0.040	0.052
Galactose	0.065	0.095
Xylose	0.113	0.214
Arabinose	0.184	0.045

CMP-H: *C. morifolium* polysaccharides obtained by hot reflux extraction; CMP-U: *C. morifolium* polysaccharides obtained by ultrasonic assisted extraction; different letters (^a,b^) in superscript for each index denote a significant difference (*p* < 0.05).

**Table 4:** Molecular weight distribution of CMP-U and CMP-H.

Molecular Weight Distribution				
Polysaccharides	Retention Time (min)	Mw (Da)	Mn (Da)	Area %
CMP-U	13.987	669,143	153,373	26.27
	18.492	2115	1231	36.21
	20.205	214	179	37.51
CMP-H	14.350	521,905	108,418	37.44
	18.557	2628	1464	28.74
	20.058	321	225	33.82

CMP-H: *C. morifolium* polysaccharides obtained by hot reflux extraction; CMP-U: *C. morifolium* polysaccharides obtained by ultrasonic assisted extraction.

## Abbreviations

CMPs: *Chrysanthemum morifolium* polysaccharides; HRE: heat reflex extraction; ROS: reactive oxygen species; DPPH: 1,1-diphenyl-2-picrylhydrazyl radical; RSM: response surface methodology; TCA: trichloroacetic acid; FT-IR: Fourier transform infrared; PMP: 1-phenyl-3-methyl-5-pyrazolone; *p*NPG: 4-nitrophenyl-β-D-glucopyranoside; BSA: bovine serum albumin; HPGPC: high performance gel permeation chromatography; HPLC: high performance liquid chromatography; BHA: butyl hydroxyanisole; TFA: trifluoroacetic acid.
